# Supplementary material for: Formal Meta-Analysis of Hypoxic Gene Expression Profiles Reveals a Universal Gene Signature
Source: Biomedicines. 2022 Sep 8;10(9):2229. doi: 10.3390/biomedicines10092229 (PMC9496516; doi:10.3390/biomedicines10092229)
Supplement: Supplementary file 1 [file biomedicines-10-02229-s001.zip › Supplementary.pdf]

biomedicines

MDPI

Article

Materials supplementary to Formal Meta-Analysis of Hypoxic Gene Expression Profiles Reveals a Universal Gene Signature

Laura Puente-Santamaria [1,2], Lucia Sanchez-Gonzalez [1], Nuria Pescador [3], Oscar Martinez-Costa [1], Ricardo Ramos-Ruiz [2] and Luis del Peso [1,4,5,6,*]

[1] Departamento de Bioquímica, Universidad Autónoma de Madrid (UAM) and Instituto de Investigaciones Biomédicas "Alberto Sols" (CSIC-UAM), 28029 Madrid, Spain
[2] Genomics Unit Cantoblanco, Fundación Parque Científico de Madrid, C/ Faraday 7, 28049, Madrid, Spain
[3] Centro de Investigación Biomédica en Red de Diabetes y Enfermedades Metabólicas Asociadas (CIBERDEM), Instituto de Salud Carlos III, Spain.
[4] IdiPaz, Instituto de Investigación Sanitaria del Hospital Universitario La Paz, 28029 Madrid, Spain
[5] Centro de Investigación Biomédica en Red de Enfermedades Respiratorias (CIBERES), Instituto de Salud Carlos III, Spain
[6] Unidad Asociada de Biomedicina CSIC-UCLM, 02006, Albacete, Spain
* Correspondence: luis.peso@uam.es

Abstract: Integrating transcriptional profiles results in identifying gene expression signatures that are more robust than those obtained for individual datasets. However, a direct comparison of datasets derived from heterogeneous experimental conditions is impossible, and their integration requires applying of specific meta-analysis t echniques. The transcriptional response to hypoxia has been the focus of intense research due to its central role in tissue homeostasis and prevalent diseases. Accordingly, many studies have determined the gene expression profile of hypoxic cells. Yet, despite this wealth of information, little effort has been made to integrate these datasets to produce a robust hypoxic signature. We applied a formal meta-analysis procedure to datasets comprising 430 RNA-seq samples from 43 individual studies including 34 different cell types, to derive a pooled estimate of the effect of hypoxia on gene expression in human cell lines grown in vitro. This approach revealed that a large proportion of the transcriptome is significantly regulated by hypoxia (8556 out of 20888 genes identified across studies). However, only a small fraction of the differentially expressed genes (1265 genes, 15%) show an effect size that, according to comparisons to gene pathways known to be regulated by hypoxia, is likely to be biologically relevant. By focusing on genes ubiquitously expressed, we identified a signature of 291 genes robustly and consistently regulated by hypoxia. Overall, we have developed a robust gene signature that characterizes the transcriptomic response of human cell lines exposed to hypoxia in vitro by applying a formal meta-analysis to gene expression profiles.

Keywords: transcription; hypoxia; RNAseq; meta-analysis

check for updates

Citation: Lastname, F.; Lastname, F.; Lastname, F. Formal Meta-Analysis of Hypoxic Gene Expression Profiles Reveals a Universal Gene Signature. *Biomedicines* **2022**, *10*, 2229. https://doi.org/10.3390/biomedicines10092229

Academic Editor: Shaker A. Mousa

Received:
Accepted:
Published:

Publisher's Note: MDPI stays neutral with regard to jurisdictional claims in published maps and institutional affiliations.

Copyright: © 2022 by the authors. Licensee MDPI, Basel, Switzerland. This article is an open access article distributed under the terms and conditions of the Creative Commons Attribution (CC BY) license (https://creativecommons.org/licenses/by/4.0/).

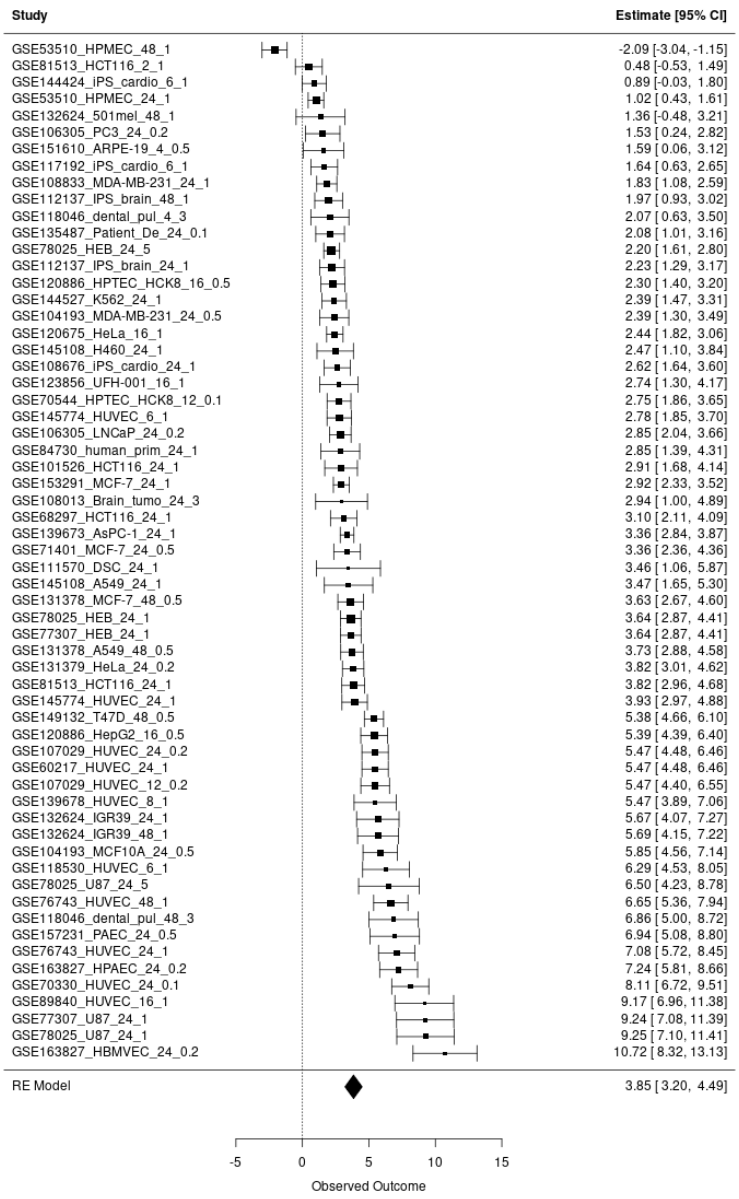

Figure S1. Meta-analysis of the effect of hypoxia on EGLN3 gene expression. Forest plot representing the individual effect size (LFC) estimates as black boxes whose size is inversely proportional to the precision of the estimate. Each estimate derives from the study indicated on the left column ("Study"), which includes information about GEO record and experimental conditions: cell line, concentration of oxygen (percentage of oxygen in gas mixture) during exposure to hypoxia and length of hypoxia in hours. The pooled effect size estimate is shown at the bottom as a black diamond. The columns on the left indicates the values of the effect size (Log_2FC), the 95% confidence interval for the estimate and the weight of each observation on the pooled effect. Pooled effect size was significantly different from zero ($p - value = 2 \times 10^{-17}$)

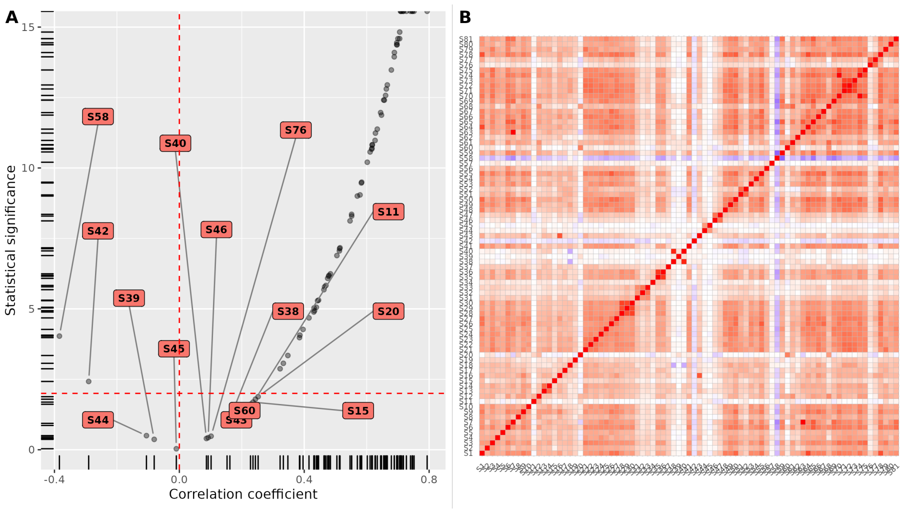

Figure S2. Identification of outlier subsets. (**A**) The pooled effect of hypoxia on gene expression was compared to the estimates obtained in each individual subset for all the genes that were ubiquitously expressed (expression detected in more than 75% of subsets) and that showed a strong ($|Log_2FC| > 1$) and significant ($FDR < 0.01$) response to hypoxia according to the meta-analyses. Each dot represents the Pearson's correlation coefficient (r) and its statistical significance (FDR-adjusted p-value). The horizontal and vertical dotted lines correspond to an adjusted p-value of 0.01 and a correlation coefficient of zero respectively. Those subsets that did not showed significant correlation with the meta-analyses pooled estimates ($FDR > 0.01$) or showed a negative correlation ($r < 0$) are identified and labeled. (**B**) Correlation between all pairs of datasets. The figure shows the Pearson's correlation coefficient for all pair-wise comparisons as a heat map. Positive values correlation coefficients are shown in red color and negative values in blue, increased color saturation is used to represent more extreme values. The color and shape of the ellipses indicate the strength of the correlation (value of Pearson's r) and its direction.

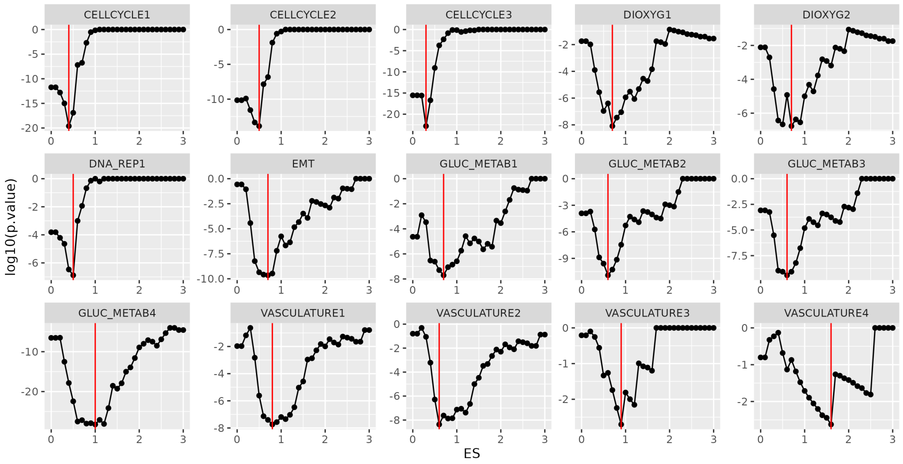

Figure S3. Identification of an effect size value that maximizes the association between gene expression and cellular responses to hypoxia. Genes showing an statistically significant change in expression ($FDR < 0.01$) of a magnitude ($|Log_2FC|$) above the indicated values (X axis) were categorized as differentially expressed in response to hypoxia. Then, genes were further classified into mutually exclusive subgroups according to their membership regarding the indicated biological processes. Finally, the association between the response to hypoxia and the biological process was assessed using a Fisher's exact test. Each graph shows the p-value for the the association between the indicated biological process (Gene Ontology Biological Processes: "DNA_DEPENDENT_DNA_REPLICATION" (CELLCYCLE1), "CELL_CYCLE_DNA_REPLICATION" (CELLCYCLE2), MSig DB Hallmark: "G2M_CHECKPOINT" (CELLCYCLE3), . . .) and the response to hypoxia at different Log_2 FC cut-off values.

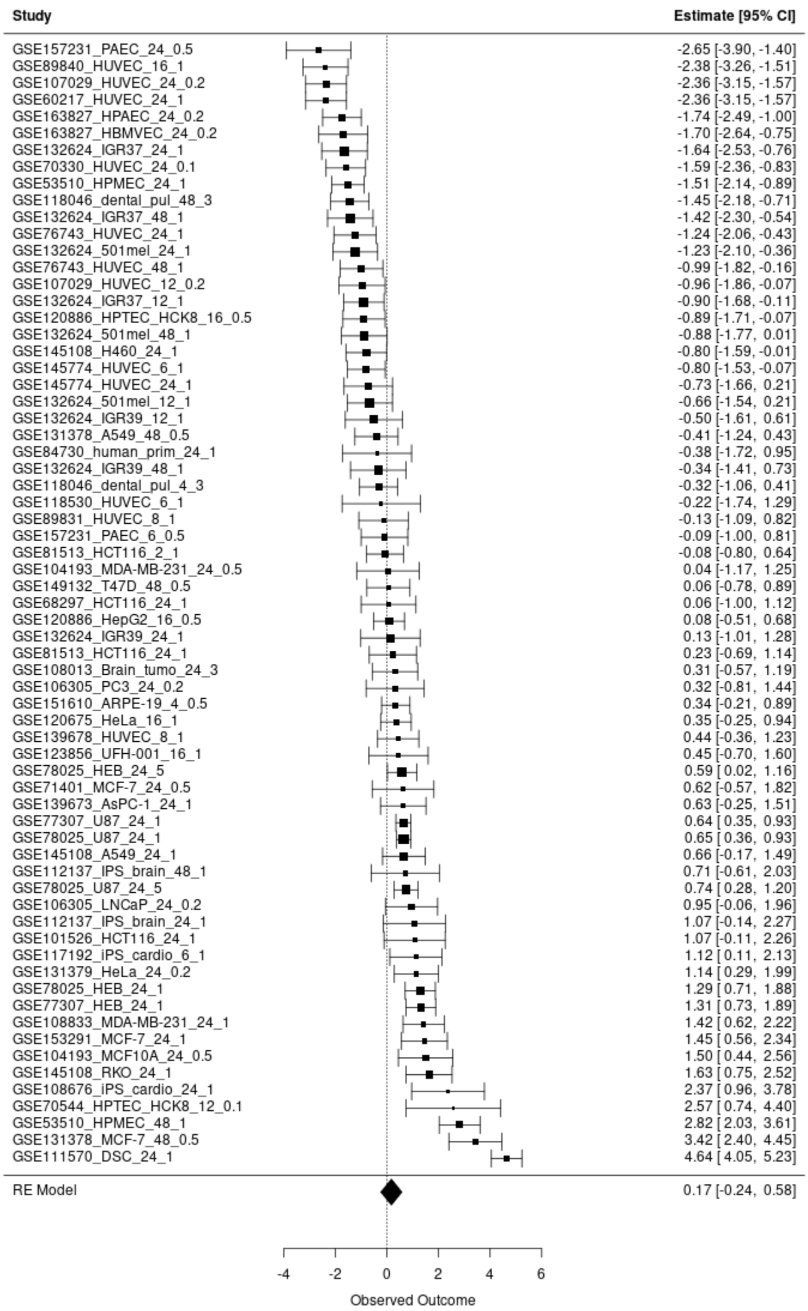

Figure S4. Meta-analysis of the effect of hypoxia on HMOX1 gene expression. Forest plot representing the individual effect size (LFC) estimates as grey boxes whose size is inversely proportional to the precision of the estimate. Each estimate derives from the study indicated on the left column ("Study"), which includes information about GEO record and experimental conditions: cell line, concentration of oxygen (percentage of oxygen in gas mixture) during exposure to hypoxia and length of hypoxia in hours. The pooled effect size estimate is shown at the bottom as a blue diamond. The columns on the left indicate the values of the effect size (Log_2FC), the 95% confidence interval for the estimate and the weight of each observation on the pooled effect.

Legends to Supplementary tables (on-line)

Table S1. Studies selected from database search. GSE_ID, GEO Series ID; The oxygen tensions hypoxia, exposure times (in hours) and cell type(s) included in the study are shown in columns O_2, Hx_time and CT respectively.

Table S2. Frequency of hypoxia-repressed genes. Gene_symbol, Human Genome Nomenclature gene symbol; N, number of subsets (out of a total of 81) where the indicated gene was found significantly down-regulated ($FDR < 0.01$ and $LogFC < 0$) by hypoxia.

Table S3. Frequency of hypoxia-induced genes. Gene_symbol, Human Genome Nomenclature gene symbol; N, number of subsets (out of a total of 81) where the indicated gene was found significantly up-regulated ($FDR < 0.01$ and $LogFC > 0$) by hypoxia.

Table S4. Metadata of studies kept after filtering original datasets. Columns 1–8 (Run, BioProject, BioSample, Experiment, Sample.Name, SRA.Study, source_name, Treatment) correspond to the information associated to samples in the NCBI's Sequence Read Archive (SRA). GSE_ID, GEO Series ID; The cell line name, cell type, oxygen tension (O_2%) and hypoxia exposure time (in hours) was extracted from individual studies and recorded in columns Cell_Line, O_2 and Hx_time respectively.

Table S5. Compiled Meta-analyses results. Gene_symbol, Human Genome Nomenclature gene symbol; N, number of Nx-Hx sample pairs (subsets) were gene expression was detectable; Beta, pooled estimate of effect size ($\log_2$ Hx/Nx); SE, Standard Error of the pooled estimate; tval, test statistics of the Beta value; df, degrees of freedom; pval, corresponding *p*-values; ci.lb, lower bound of the confidence intervals for Beta; ci.ub, lupper bound of the confidence intervals for Beta; ajdP, FDR-adjusted *p*-values.

Table S6. Biological pathways repressed by hypoxia. List of pathways repressed by hypoxia according to GSEA. ES, Enrichment Score; NES, Normalized Enrichment Score; NOM.p.val, Nominal *p*-value, statistical significance of the enrichment score; FDR.q.val, FDR-corrected *p*-value; FDR.q.val, FWER-corrected *p*-value; RANK-AT-MAX, position in the ranked list at which the maximum enrichment score occurred; LEADING.EDGE, indicates the percentage of genes in the geneset above or below the maximum ES (tags), the percentage of genes in the ranked set above or below the maximum ES (list) and a statistic combining both (signal).

Table S7. Biological pathways induced by hypoxia. List of pathways repressed by hypoxia according to GSEA. ES, Enrichment Score; NES, Normalized Enrichment Score; NOM.p.val, Nominal *p*-value, statistical significance of the enrichment score; FDR.q.val, FDR-corrected *p*-value; FDR.q.val, FWER-corrected *p*-value; RANK-AT-MAX, position in the ranked list at which the maximum enrichment score occurred; LEADING.EDGE, indicates the percentage of genes in the geneset above or below the maximum ES (tags), the percentage of genes in the ranked set above or below the maximum ES (list) and a statistic combining both (signal).

Table S8. Effect of hypoxia on the expression of the genes identified in the meta-analysis as the core hypoxic signature. Columns 1–5 as in Table S5. Remaining columns indicate the effect of hypoxia (log_2 Hx/Nx) on the gene indicated in column Gene_Symbol as determined in the study indicated in the column name. Study column names contain the following four pieces of information: GSE ID, cell type, exposure time (in hours) and percentage of oxygen separated by underscores.

Table S9. Comparison of Meta-analysis derived core genes and Hall Mark hypoxia signature. MA, Meta-analysis; HM, MSig's Hypoxia Hall Mark; Size, number of genes in the signature; N, median number of subsets were genes are present; LFC, median Log_2 Fold Change.

Table S10. Effect of hypoxia on the expression of the genes included in gene signatures. Columns 1–4 as in Table S5. Columns "Overlap" indicates whether the gene (row) is present in the Meta-analysis and MSigDB's Hall Mark gene signatures (MA_and_HM) or only in the latter (HM_only).
